# What influences recruitment to randomised controlled trials? A review of trials funded by two UK funding agencies

**DOI:** 10.1186/1745-6215-7-9

**Published:** 2006-04-07

**Authors:** Alison M McDonald, Rosemary C Knight, Marion K Campbell, Vikki A Entwistle, Adrian M Grant, Jonathan A Cook, Diana R Elbourne, David Francis, Jo Garcia, Ian Roberts, Claire Snowdon

**Affiliations:** 1 Health Services Research Unit, University of Aberdeen, Polwarth Building, Foresterhill, Aberdeen, UK; 2 Medical Statistics Unit, London School of Hygiene and Tropical Medicine, Keppel Street, London, UK; 3 Centre for Research and Innovation Management, Brighton

## Abstract

**Background:**

A commonly reported problem with the conduct of multicentre randomised controlled trials (RCTs) is that recruitment is often slower or more difficult than expected, with many trials failing to reach their planned sample size within the timescale and funding originally envisaged. The aim of this study was to explore factors that may have been associated with good and poor recruitment in a cohort of multicentre trials funded by two public bodies: the UK Medical Research Council (MRC) and the Health Technology Assessment (HTA) Programme.

**Methods:**

The cohort of trials was identified from the administrative databases held by the two funding bodies. 114 trials that recruited participants between 1994 and 2002 met the inclusion criteria. The full scientific applications and subsequent trial reports submitted by the trial teams to the funders provided the principal data sources. Associations between trial characteristics and recruitment success were tested using the Chi-squared test, or Fisher's exact test where appropriate.

**Results:**

Less than a third (31%) of the trials achieved their original recruitment target and half (53%) were awarded an extension. The proportion achieving targets did not appear to improve over time. The overall start to recruitment was delayed in 47 (41%) trials and early recruitment problems were identified in 77 (63%) trials. The inter-relationship between trial features and recruitment success was complex. A variety of strategies were employed to try to increase recruitment, but their success could not be assessed.

**Conclusion:**

Recruitment problems are complex and challenging. Many of the trials in the cohort experienced recruitment difficulties. Trials often required extended recruitment periods (sometimes supported by additional funds). While this is of continuing concern, success in addressing the trial question may be more important than recruitment alone.

## Background

Randomised controlled trials (RCTs) are widely accepted as the gold standard for evaluating healthcare interventions [1,2] and decision makers are increasingly looking to the results of RCTs to guide practice. RCTs are a major, increasing component of both NHS-supported and non-commercially funded research[3]. Recruitment is often slower or more difficult than expected, with many trials failing to reach their planned sample size within the timescale and funding originally envisaged. If the target sample size is not achieved, results will usually be less reliable. If recruitment has to be extended to reach the required sample size, this usually costs more and the use of the results in clinical practice will be delayed.

The reasons why certain trials recruit well while others do not remain unclear[4]. To investigate this the UK NHS R&D National Methodology Programme and the UK Medical Research Council (MRC) funded a project – Strategies for Trials Enrolment and Participation Study (STEPS) considering three aspects of recruitment: a review of a cohort of trials funded by both organisations; case studies of trials which appeared to have particularly interesting lessons for recruitment (exemplars); and an in-depth case study of one large multicentre trial to examine the feasibility of applying a business-orientated analytical framework. This paper describes the review of the cohort of trials, which explored factors that may have influenced recruitment and concentrated on issues relating to the researchers and clinicians conducting the trials. Full details of the project are published elsewhere[5].

The objectives of the review were to:

• characterise the trials in terms of the settings, interventions and outcomes studied;

• characterise the factors thought likely to affect success of recruitment including level of funding, complexity of trial design, involvement of a trials support unit etc;

• describe patterns of recruitment (such as to original plan or better, slower start than anticipated, recruitment with identifiable change-points etc); and

• note trialists' reports of factors perceived to be associated with good or poor recruitment (eg delays in obtaining funding or research ethics approval) and strategies attempted to improve recruitment.

## Methods

### Trial identification

Trials were identified from the administrative databases held by the two funding bodies. Trials were eligible for inclusion if:

• they involved more than one clinical centre;

• recruitment started on or after 1 January 1994 (this cut-off was chosen as the HTA Programme was established during 1993); and

• recruitment was originally planned to close on or before 31 December 2002. Trials that were awarded an extension to the recruitment phase beyond 31 December 2002 were included if they had closed to recruitment at the time of data extraction.

Cluster trials were excluded as recruitment issues are often different compared with individually randomised trials[6].

### Development of hypotheses

*A priori* hypotheses were developed about factors that might affect recruitment, related to issues in previous literature reviews [4] and insights gained from the MRC clinical trials enquiry[7].

### Data extraction

Application forms and progress reports were examined at the central offices of the two funders following notification to the appropriate principal investigators (PIs). Access was subject to confidentiality safeguards.

A data extraction form was developed to facilitate systematic collection of information under six main topic headings: 1) trial identifying details; 2) trial administrative details (eg dates of commencement, start of recruitment); 3) trial features (eg if there was a formal pilot phase, if there was a dedicated trial manager); 4) finance; 5) recruitment summary (eg original target, any revisions, final recruitment numbers); and 6) description of delay or failure to reach recruitment targets (eg any delays to recruitment, strategies used to improve recruitment). The data extraction form and procedures were piloted on eight trials. Minor modifications were made before proceeding.

Where information was not recorded or unclear, reports of the specific trials held on the Current Controlled Trials meta-register of randomised trials  and the NCCHTA website  were searched to augment the dataset. Some PIs and trial managers were also asked to provide additional information.

## Results

One hundred and fourteen trials fulfilled the inclusion criteria. Forty-one (36%) trials were funded by the HTA Programme and 73 (64%) by the MRC. Five of these, although presented under single project banners, were found to represent a number of sub-trials, each with their own individual sample size targets and individual recruitment issues. For the purposes of describing the trial features, the denominator is based on the number of recorded 'projects', ie 114, to reflect the fact that trial teams were common across sub-trials. For the analysis of recruitment issues, however, we treated the sub-trials separately, using the denominator of 122 trials.

### Overall trial features

Table 1 describes features of the trials. Sixty trials (53%) had conducted a formal pilot study. Of these, 32 indicated the pilot study had led to a change in recruitment strategy for the main trial. The most common changes noted were that: written trial materials were modified (8 trials); the trial design was changed (6 trials); changes were made to the inclusion criteria (4 trials); the recruitment target was changed (4 trials); and, the number of sites was increased (4 trials). Most trials (78%) were conducted from a trials unit and 92% involved multidisciplinary teams. No trial had a consumer as a grant applicant although 9 trials had some form of consumer involvement during the trial.

**Table 1:** Descriptive features of trials and disciplines represented amongst trial investigators

**Trial feature**	**Valid N**	**Yes** ** n (%)**	**No** ** n (%)**	**Not known** ** n (%)**
Trial had pilot study	114	60 (53)	41 (36)	13 (11)
•*Was pilot funded?*	60	35 (58)	4 (7)	21 (35)
•*Was there a change in recruitment strategy because of pilot study?*	60	32 (53)	5 (8)	23 (39)
Trial co-ordinated from trials unit	114	89 (78)	25 (22)	
Trial had dedicated trial manager	114	86 (75)	14 (12)	14 (12)
Trial had paid local staff available	114	61 (54)	31 (27)	22 (19)
Disciplines	113*			
*Multidisciplinary*		104 (92)	9 (8)	
*Consumer*		-	113 (100)	
*Economics*		66 (58)	47 (42)	
*HSR*		35 (31)	78 (69)	
*Medical/Dentist*		109 (96)	4 (3)	
*Nursing*		22 (19)	91 (80)	
*Statistics*		83 (73)	30 (26)	
*Other*		17 (15)	96 (85)	

* Data was missing for one trial

Funding ranged from £16 per planned participant to £4522 with a median of £641. The trials in our cohort started recruitment during the period 1994–2002. During this time, most trials only included research costs. However for some early trials treatment/support costs were not distinguishable from research costs due to the costing structure used for grants at that time. Twenty-four (24/89, 29%) trials were awarded a 'good' level of funding (defined as more than £1000 per planned participant, definition generated through consensus with the project management group members).

### Individual trial features

Table 2 summarises the characteristics of the 122 trials, including design features, clinical area, setting and interventions. The majority of the trials were simple parallel group trials (113, 93%). Most were two-arm (94, 77%) trials, mainly representing cancer (25, 20%), mental health (21, 17%) and orthopaedics/rheumatology (21, 17%). Approximately half (64, 53%) of the trials were in a hospital setting.

**Table 2:** Characteristics of the trials

**Characteristic**	**N**	**n (%)**
***Design:***	122	
Parallel		113 (93)
Factorial		6 (5)
Partially randomised patient preference		3 (2)
***Number of arms:***	122	
Two		94 (77)
Three		18 (15)
More than three		10 (8)
***Clinical areas:***	122	
Cancer		25 (20)
Mental health (including neurosciences/psychiatry/psychology)		21 (17)
Orthopaedics/rheumatology (including back pain)		21 (17)
Obstetrics & Gynaecology		9 (7)
Primary care		8 (7)
Cardiology		5 (4)
Gastroenterology		5 (4)
Incontinence/urology		5 (4)
HIV/AIDS		5 (4)
Other		18 (15)
***Setting:***	122	
Hospital		64 (53)
General practice		26 (21)
Mixed		16 (13)
Community		7 (6)
Missing		9 (7)
***Geographical spread:***	122	
Multiple regions		63 (52)
Regional		51 (42)
Missing		8 (7)
***Any recruiting centres outside UK****:*	114	
No		88 (77)
Yes		25 (22)
Missing		1 (1)
***Interventions:***	122	
Medical (drugs, injections) excluding chemotherapy		37 (30)
Behavioural therapies (eg CBT with or without conventional drugs)		12 (10)
Different types of surgical intervention (including laparoscopic)		12 (10)
Chemotherapy		10 (8)
New services/treatment policy/information provision (eg support programmes)		9 (7)
Radiology (including ultrasound)		8 (7)
Medical instruments (eg metal stents, pacemakers, bandage types)		7 (6)
Surgery v alternative (eg conservative management, radiotherapy)		4 (3)
Alternative therapies (including complementary medicine, water-based therapies)		4 (3)
Other		19 (16)

### Recruitment

Recruitment targets varied from 60 to 66,000 participants. Table 3 summarises actual recruitment in relation to these targets. Thirty-eight (31%) trials 'successfully' recruited (≥ 100% of their original target). A further 29 (24%) trials achieved a recruitment rate of greater than 80% but less than 100% of their original target.

**Table 3:** Recruitment in trials

	**N**	**n (%)**
***Recruited successfully***	122	
Yes		38 (31.1)
No		84 (68.9)
***Was recruitment target revised***	*122*	
Yes		42 (34.4)
No		76 (62.3)
Missing		4 (3.3)
***Final recruitment figure***		
*Original target:*	122	
≥ 100%		38 (31.1)
≥ 80% but < 100%		29 (23.8)
< 80%		55 (45.1)
*Revised target:*	42	
≥ 100%		19 (45.2)
≥ 80% but < 100%		15 (35.7)
< 80%		8 (19.1)

In 42 (34%) trials the recruitment target was revised during the trial. The target was revised in a downward direction in 36 (86%) of the 42. Only 19 (19/42, 45%) trials whose targets were revised were known to have successfully recruited to their new target.

Enrolment was halted before the formal end of the recruitment period in 14 (14/122, 11%) trials. In 11 this decision related to poor recruitment. In the three others, early termination followed a recommendation from a data monitoring committee that there were clear differences between the trial groups. However, in two of these, the recruitment period had already been extended beyond that originally specified.

Sixty-six (54%) trials requested an extension to the trial grant to complete the original trial and, in all but one, either a time plus grant extension (42, 64%), a time-only extension (15, 23%) or a supplementary grant only (8, 12%) was awarded. Thirteen trials recruited their original target after a time extension.

**Figure 1:**
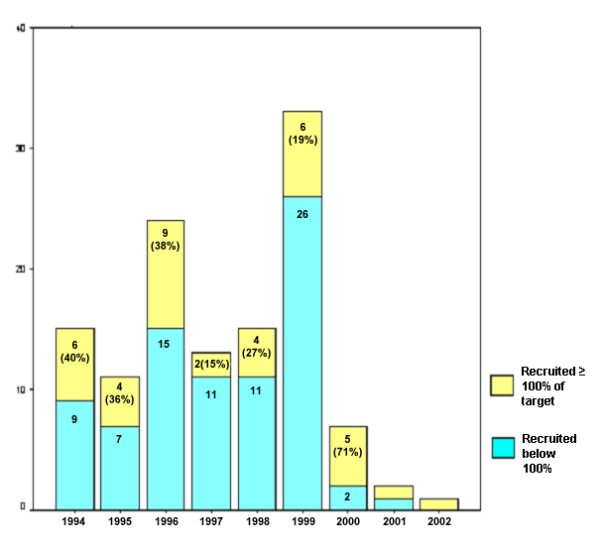
Recruitment success related to year trial commenced.

Figure 1 describes recruitment success by year of trial commencement. Six of the fifteen trials (40%) that commenced recruitment in 1994 recruited to, or above, 100% of their original recruitment target. Lower proportions of trials achieved their targets amongst those started in the late 1990s.

### Delays to recruitment

The start of recruitment was delayed in 47 (41%) of trials (data not shown). Main reasons cited were: delays related to central trial staff (11 trials); local research staff (11 trials); and local clinical arrangements (seven trials). Other reasons identified included: delays with ethics; supply of study drugs/placebo; development of clinical guidelines impacting on the trial; PI moving; adverse publicity about research; and publication of conflicting research.

Eighty six (75%) trialists indicated at the application stage that they had pre-identified trial centres. In 17 cases some pre-identified centres did not participate as planned. There was no common reason for this, although the issue of problems with "treatment" and "service support" costs were raised in three cases. Thirty-seven (32%) trialists indicated that they encountered delay in bringing in some of the pre-identified centres. Reported reasons included: problems with costs/funding (13 trials); delays in the recruitment of staff (12 trials); and changes with the Multicentre Research Ethics Committee (MREC) system (6 trials). Fifty-two (45%) trials recruited new centres to ensure the delivery of the trial.

Once centres were enrolled, early recruitment (within the first 25% of scheduled recruiting time) was reported to be slower than anticipated in 77 (63%) trials. Common reasons for this were: fewer eligible patients than expected (19 trials); internal problems eg with staff (18 trials); and a smaller percentage of patients agreeing to participate than was expected (16 trials). Other problems included: eligible patients missed (10 trials); external problems eg publicity (8 trials); funding issues (5 trials) and issues with procedures/interventions eg randomisation or placebo (5 trials).

Delays to later recruitment (within the last approximate 75% of recruiting time) were reported in 46 (38%) trials. The most common reasons noted in this phase were: internal problems eg staff (10 trials); fewer patients agreeing to participate than expected (9 trials); fewer eligible patients than anticipated (7 trials); and external problems, particularly with publicity (7 trials). Numerous other problems included: funding difficulties (5 trials); conflicts with other trials (5 trials); and long waiting lists (3 trials).

### Relationships between trial features and successful recruitment

Trial features that were pre-specified as likely to enhance the chances of successful recruitment are presented in Table 4. An association suggesting that the factor increased successful recruitment is indicated by an odds ratio (OR) of greater than unity. Only trials for which the information was known were included. Confidence intervals (CIs) around the OR estimates were wide, reflecting the maximum sample size (122). Some of the comparison cells had very few data (eg number with a complex design and number with consumer input). There were marginally statistically significant associations with MRC funding, being a cancer trial, and with not having paid local trial coordinators.

**Table 4:** Associations between features and recruitment success

**Feature**	**Valid N**	Trials with feature that recruited successfully* **n (%)**	Trials that didn't have feature that recruited successfully* **n (%)**	**Odds Ratio**	**95%CI**	**p-value**
Local recruitment coordinators	100	15/69 (22)	14/31 (45)	0.34	0.14, 0.84	0.017
Simple design	122	35/116 (30)	3/6 (50)	0.43	0.06, 3.41	0.374
Support from a trials unit	122	27/94 (29)	11/28 (39)	0.62	0.26, 1.50	0.289
Multidisciplinary input	113	34/104 (33)	3/9 (33)	0.97	0.19, 6.36	0.615
Pilot phase	109	18/66 (27)	11/43 (26)	1.09	0.46, 2.61	0.845
Good level of funding	89	7/24 (29)	17/65 (26)	1.16	0.41, 3.29	0.776
Drug trial	122	19/53 (36)	19/69 (28)	1.47	0.68, 3.18	0.326
Interventions only available inside the trial	112	7/18 (39)	26/94 (28)	1.66	0.58, 4.76	0.338
Consumer input	107	4/9 (44)	26/91 (29)	2.00	0.36, 10.05	0.446
Funded by the MRC	122	28/74 (38)	10/48 (21)	2.31	1.00, 5.36	0.048
Cancer trial	122	12/24 (50)	26/98 (27)	2.77	1.11, 6.93	0.026
Dedicated trial manager	107	32/91 (35)	2/16 (13)	3.80	0.79, 36.14	0.087

* ≥ 100% of original target

### Reported strategies for improving recruitment

Seventy-three trials reported using a variety of strategies to improve recruitment (Table 5). The most common was the use of newsletters and mail shots, both to clinical staff and patients. Ten per cent of the trials reported that inclusion criteria were changed or the protocol amended to improve recruitment.

**Table 5:** Most commonly reported strategies to improve recruitment (N = 122)

**STRATEGY**	**No. trials**
Newsletters/mail shots/flyers (to clinical staff and/or patients)	26
Regular visits/phone calls to wards/sites/practices	15
Posters/information leaflets in clinics/wards/notes	13
Inclusion criteria changed/protocol amended	12
Presentations to appropriate groups eg at consultant meetings/community based physiotherapists etc	10
Resource manual for site staff/trained staff in disease area/procedures being investigated/role play exercises/study day/workshops for recruiters	10
Advertisement/articles in newspapers/journals; radio interviews	8
Presentations at national/international meetings	6
Employed extra staff	6
Investigators'/recruiting staff meetings	5
Training/information videos	4
Incentives for recruiters eg prize draw, chocolates etc	4
Trial material revised/simplified/customised for specific sites	4
Visits to centres by PIs/senior members of study group	3
Repeated contact by phone/letter to individuals/sites	3
Increased/changed time points when information provided to potential participants	3
Supportive statements from opinion leaders	3

Note Only those reported by 3 or more trials are listed

## Discussion

Data from two historical cohorts of trials has shown that failure to achieve projected targets for recruitment has been common amongst multicentre trials supported by the main funders of trials in the UK.

Fewer than one third recruited to 100% of their original target, and 45% failed to recruit to within 80% of target. Factors associated with successful recruitment were that one or more intervention was only available inside the trial, having a dedicated trial manager, and being a cancer or a drug trial. These findings should be interpreted cautiously; CIs were wide, associations were only marginally statistically significant for some variables, and the trend for some factors was towards a negative association. Commonly reported strategies to improve recruitment were newsletters and mail shots, but it was not possible to assess whether they were causally linked to changes in recruitment.

The anticipated recruitment period was extended for around half of trials, usually supported by a supplementary grant. Delays were experienced at all stages of recruitment. The most commonly reported problem with early recruitment was that fewer than expected eligible patients were being observed. This phenomenon, known as "Lasagna's Law" – where researchers and clinicians invariably overestimate the number of patients available for study participation – has been observed by a number of commentators[8]. Factors thought to be potentially associated with successful recruitment were relatively uninformative. The CIs around the estimated ORs were all wide and too imprecise to allow judgement about possible causal relationships.

A major strength of this study was that it included a systematically identified, complete cohort of trials funded by the two major UK public funding bodies in the field of health care and drew on previously confidential routine progress reports submitted over the course of a trial by the investigators. The trials represented a wide spectrum of clinical areas, clinical settings and geographical centres. However, participants' perceptions of what makes a trial attractive to join could not be elicited. The results of our study complement the more in-depth qualitative investigations which have tried to specifically address participants' perceptions[9-11].

Recruitment can be viewed as a surrogate measure of other, less easily quantifiable, but arguably more significant measures of trial success, such as 'impact on clinical practice', or the extent to which the trial question has been addressed. Other parameters should also be taken into account, such as rates of participant retention and treatment compliance.

We hoped to identify factors associated with successful recruitment to provide a means of predicting or enhancing the chances of success. However, comparative analyses yielded few insights, because of the choice of outcome and exposure variables, and because of imprecision around the estimates of association. Some factors (such as the intervention only being available inside the trial, having a dedicated trial manager, and being a cancer trial or a drug trial) may be associated with successful recruitment, these results were also (with the possible exception of cancer trials) compatible with there being no association. Other analyses showed that some features expected to enhance recruitment were less commonly observed in 'successful' trials than in unsuccessful trials. This applied in particular to 'local paid recruitment coordinators', but an alternative explanation for this negative association is that the relationship comparison is confounded by other factors such as the complexity of the trial and the years when it was undertaken. As many of the variables are potentially correlated a multivariable analysis would have been desirable. We did not have sufficient power to undertake this analysis – experts suggest that there should be at least ten observations in the dataset for each potential explanatory variable to be included in the model[12].

As indicated above, there was evidence that cancer trials were associated with better rates of successful recruitment compared with non-cancer trials. The National Cancer Research Network (NCRN) was established by the Department of Health in April 2001 to improve the infrastructure within the NHS for clinical research in cancer in England (most of the trials included in our study had completed recruitment before this initiative had been established). There has been a doubling of the recruitment rate to cancer trials since the inception of the NCRN[13]. Building on this success, the recently formed UK Clinical Research Network  will continue the aim of improving the speed, quality and integration of research, initially within the existing NHS networks in cancer and mental health and new ones in the priority areas of medicines for children, stroke, diabetes and Alzheimer's disease.

## Conclusion

This study highlights the challenges of recruiting to RCTs and provides a valuable overview of trialists' attempts to overcome them. Despite the growing literature summarising barriers and facilitators to recruitment published in the 1990s (exemplified by the HTA review by Prescott [4]) only 55% of the trials recruited to within 80% of the original target and the situation does not seem to have improved over time. It is clear that recruitment to trials is a complex problem. The two other components of STEPS considering lessons from exemplar trials and from a business perspective will be reported elsewhere.

## Competing interests

MC, DE, IR, JG, AG and AM all hold research grants for multicentre trials from the MRC and, or, the HTA Programme. AM and RK have received salary support from MRC trial grants in the past.

## Authors' contributions

All STEPS Group members contributed to the project – the idea for the study was jointly conceived by the Principal Investigator, Marion Campbell (MKC) (guarantor), with Adrian Grant (AMG), Vikki Entwistle (VAE), Diana Elbourne (DE), Jo Garcia (JG), Claire Snowdon (CS), Ian Roberts (IR) and David Francis (DF). Alison McDonald (AMM) and Rosemary Knight (RCK), together with the other STEPS members, helped to design the study and did the data extraction. Jonathan Cook (JAC) undertook the analysis and, together with all group members, interpretation of data. All authors contributed to the final manuscript.
